# Endothelial exosomes work as a functional mediator to activate macrophages

**DOI:** 10.3389/fimmu.2023.1169471

**Published:** 2023-07-28

**Authors:** Wenwen Lin, Feng Huang, Yin Yuan, Qiaowei Li, Zhong Lin, Wenqing Zhu, Binbin Lin, Pengli Zhu

**Affiliations:** ^1^ Department of Geriatric Medicine, Fujian Provincial Hospital, Fuzhou, China; ^2^ Shengli Clinical Medical College, Fujian Medical University, Fuzhou, China; ^3^ Fujian Provincial Key Laboratory of Geriatrics, Fuzhou, China; ^4^ Fujian Provincial Institute of Clinical Geriatrics, Fuzhou, China; ^5^ Fujian Provincial Center of Geriatrics, Fuzhou, China

**Keywords:** exosomes, tumor necrosis factor-α, endothelial cell, macrophage, atherosclerosis

## Abstract

**Introduction:**

Intercellular communication is essential for almost all physiological and pathological processes. Endothelial cell (EC)-derived exosomes, working as mediators for intercellular information exchange, are involved in the pathophysiological mechanisms of atherosclerosis. However, the effect of inflamed endothelial exosomes on the function of macrophages (Mϕ) is poorly defined. This study aims to unravel how exosomes derived from tumor necrosis factor-α (TNF-α)-stimulated ECs (exo-T) affect Mϕ *in vitro*.

**Methods and results:**

Exosomes derived from untreated ECs (exo) and exo-T were identified by using TEM, NTA, and western blot, and we observed that PKH67-labeled exo/exo-T were taken up by Mϕ. Exposure to exo-T for 24 h not only skewed Mϕ to the M1 subtype and exacerbated lipid deposition, but also promoted Mϕ apoptosis, while it did not significantly affect Mϕ migration, as detected by RT-qPCR, Dil-ox-LDL uptake assay, flow cytometry, wound healing assay, and transwell assay, respectively. In addition, exo/exo-T-related microRNA-Seq revealed 104 significantly differentially expressed microRNAs (DE-miRNAs). The target genes of DE-miRNAs were mainly enriched functionally in metabolic pathways, MAPK signaling pathway, etc., as determined using Gene Ontology (GO) and Kyoto Encyclopedia of Genes and Genomes (KEGG) pathway analyses. We further demonstrated by immunoblotting that exo-T intervention improves the phosphorylation of MAPK/NF-κB-related proteins.

**Discussion and conclusion:**

Collectively, this study reveals that inflamed endothelial exosomes (TNF-α-stimulated EC-derived exosomes) work as a functional mediator to affect Mϕ function and may activate Mϕ through MAPK/NF-κB signaling pathways.

## 1 Introduction

Atherosclerosis, a chronic inflammatory vascular disease, is regarded as the pathophysiological basis of cardiovascular and cerebrovascular diseases (1). Even though many treatments have been administered in clinic, including pharmacologic therapy and exercise intervention, the mortality rate of atherosclerosis still remains considerable (2).

Presently, “Immune Inflammation Theory” is thought to be one of the mainstream pathogeneses of atherosclerosis, which involves multiple immune–inflammation intercellular interactions (3, 4). Among them, endothelial dysfunction is recognized as the most important initiation step of atherosclerosis. With endothelial dysfunction, circulating monocytes cling to activated endothelium and migrate into the subendothelial space where they differentiate into macrophages (Mϕ) (5, 6), which constitute the primary immune cell capable of balancing the pro-inflammatory and pro-resolving immune responses. Thus, the interaction between Mϕ and endothelial cells (ECs) is considered as one of the earliest initiators of the vascular inflammatory cascade, their cell-to-cell communication serving as the important functional coordination in atherosclerosis.

Recent studies have identified a novel mechanism of intercellular communication mediated by the release of exosomes. Exosomes are extracellular vesicles of 40–160 nm in diameter with a phospholipid‐bound lipid bilayer membrane structure (7), which are secreted by virtually all cells and stably exist in bodily fluids. Exosomes carry bioactive molecules such as RNA, DNA, proteins, and lipids to recipient cells and directly activate target cells, serving as an endogenous delivery vehicle for cell-to-cell communication (8).

The drumbeat of concern about endothelial-derived exosomes has grown louder in recent years. Beside presenting in healthy individuals, increased endothelial-derived exosome levels are found in conditions associated with inflammatory vascular disease, such as atherosclerosis, diabetes, sepsis, etc. (9, 10), and serve as circulating response biomarkers (11). Of note, research suggests that a multitude of bioactive factors or hemodynamic alterations can activate endothelium, thereby affecting endothelial-derived exosome contents and functions (12). And tumor necrosis factor-α (TNF-α), a pro-inflammatory cytokine, is considered as an effective irritant, leading to the formation of endothelial exosomes in the inflammatory microenvironment (13). A previous study indicated that exosomes from TNF-α-treated ECs containing a high content of calcium and BMP-2 are able to induce calcification and osteogenic differentiation of VSMCs (14). Hosseinkhani et al. (15) discovered that exosomes from TNF-α-induced ECs loaded with inflammatory markers, chemokines, and cytokines establish cross-talk between ECs and monocytes. However, infiltrating macrophages are also major contributors to cell–cell communication in the atherosclerosis microenvironment (16) (17), thus our study investigated the effects of exosomes from TNF-α-stimulated ECs on macrophages and the possible mechanism of action.

## 2 Material and methods

### 2.1 Antibodies and reagents

The following primary antibodies were used in this study: phospho-IκB-α (S32/S36) polyclonal antibody, phospho-NFκB-p65 (S536) polyclonal antibody, phospho-NFκB-p105/p50 (S337) polyclonal antibody, NFκB-p105/p50 polyclonal antibody, p38 polyclonal antibody, phospho-p38 (T180/Y182) polyclonal antibody, JNK1/2/3 polyclonal antibody, phospho-JNK1/2/3 (T183/Y185) polyclonal antibody; they were purchased from ImmunoWay Biotechnology (Plano, TX, USA). NF-κB p65 polyclonal antibody and IκB alpha polyclonal antibody were purchased from Wuhan Sanying Biotechnology Inc. (Wuhan, China). The exosome marker protein assay kit (TSG101, CD63) was purchased from Umibio (Shanghai, China). The main reagent required for the experiment, phorbol 12-myristate 13-acetate (PMA), was purchased from the biotechnology company Sigma (St. Louis, MO, USA). Human TNF-alpha was purchased from PeproTech Inc. (Rocky Hill, NJ, USA). Exosome-depleted FBS Media Supplement was purchased from SBI System Biosciences (Mountain View, CA, USA).

### 2.2 Cell culture

THP-1 cells were cultured in RMPI-1640 supplemented with 10% FBS and 1% P-S, and logarithmically growing cells were collected to be differentiated into Mϕ using 100 ng/ml PMA for 48 h. Human umbilical venous ECs were maintained in the EC medium. When ECs were grown to 70–80% confluence, they were inflammatorily stimulated by adding 10 ng/ml TNF­α in refresh medium with 10% exosome-depleted FBS for 24 h. Then, the media were collected for exosome isolation.

### 2.3 Isolation and characterization of exosomes

Exosomes were isolated from the supernatants with or without TNF­α incubation (marked as exo-T or exo) using an Exosome Isolation Kit according to instructions (Umibio, Shanghai, China). Briefly, multiple centrifugation steps were done at 3000 g for 10 min at 4 °C to remove cellular debris. Then, the supernatants were mixed with kit reagents (ESC) and incubated for 2 to 24 h at 4 °C. The supernatant was then centrifuged at 10,000 *g* at 4°C for 1 h to precipitate exosomes. The exosomes were resuspended in 1× PBS and centrifuged at 12,000 *g* for 2 min at 4°C to precipitate exosomes again. Finally, the exosomes transferred to a purification column were purified, centrifuged at 3000 g for 10 min at 4°C, resuspended in PBS, and immediately stored at −80°C until further study. (Re-extraction of exosomes using the exosome extraction protocol to remove possible residual TNF-α.) The morphology of exosomes was observed by transmission electron microscope (HT-7700, Hitachi, Tokyo, Japan) at 100 kV after 10 μl of exosomes treated with uranium acetate was dropped onto a copper grid. The size distribution and concentration of exosomes were analyzed by nanoparticle tracking analysis (NTA; N30E, NanoFCM, Xiamen, China) after exosome samples were diluted with PBS to the desired concentration. Expression of the exosomal surface marker proteins CD63 and TSG101 was identified by western blot analysis.

### 2.4 Exosome labeling and uptake

To monitor exosome trafficking *in vitro*, isolated exosomes were labeled with the green fluorescent dye PKH67 (Umibio, Shanghai, China) according to the manufacturer’s instructions. Macrophages were co-cultured with exosomes labeled with PKH67 as mentioned above for 4 h and then the cell nuclei were incubated with Hoechst (Solaibao, Beijing, China) for 5 min at 37°C. The images were captured using a High Content Imaging System (PerkinElmer, Waltham, MA, USA).

### 2.5 Quantitative real-time PCR

Total RNA was obtained using an RNA extraction kit (Bioer Technology Co. Ltd., Hangzhou, China) according to the manufacturer’s protocol, and complementary DNA was generated using a cDNA synthesis kit (Novoprotein Technology Co. Ltd., Suzhou, China). The primers are shown in (**Supplementary Table 1**). PCR amplification was conducted with SYBR qPCR SuperMix (Novoprotein Technology Co. Ltd., Suzhou, China) in an RT‐PCR system. GAPDH served as the normalization control and the relative expression of mRNA was calculated using the 2^−ΔΔCt^ method.

### 2.6 Uptake of Dil-ox-LDL into macrophages

The Dil-ox-LDL uptake assay was conducted according to the manufacturer’s protocol (Yiyuan Biotechnology Co. Ltd., Guangzhou, China). Briefly, after macrophages were treated with exo or exo-T for 24 h, and the same volume of PBS as a control, the macrophages were incubated in medium with 30 μg/ml Dil-ox-LDL for 4 h and then stained with Hoechst for 5 min at 37°C in 5% CO_2_. The fluorescence intensity was quantified by Harmony 4.5 software and a High Content Imaging System.

### 2.7 Detection of apoptotic cells

Apoptosis was assessed using an FITC AnnexinV Apoptosis Detection Kit (Dalian Meilun Biotechnology, Dalian, China) according to the manufacturer’s protocol. Briefly, the cell suspension was collected and washed with cold PBS twice. Then cells were double-stained with annexin V and propidium iodide. The percentage of apoptotic cells was evaluated by flow cytometry (FACS Accuri C6, BD Bioscience, San Jose, CA, USA).

### 2.8 Wound healing and transwell assay

Macrophages were cultured in medium with 10% exosome-depleted FBS on 12-well plates and scratch wounds were created using a 200 μl pipette tip per well before the PBS/exo/exo-T was added. The initial wound size of macrophages was measured immediately after washing the cells twice. After 24 h, images were captured again in the same fields. The area of migration was calculated as the percentage wound closure using ImageJ. The transwell assay was performed in 8 μm pore-size inserts (Corning Incorporated, Corning, CA, USA). THP-1 cells differentiated into macrophages and were cultured in the apical chamber. After 24 h of coculture with PBS/exo/exo-T in the incubator, the migratory macrophages at the bottom of the chamber were stained with DAPI (Dalian Meilun Biotechnology, Dalian, China) after fixation and were visualized using a High Content Imaging System.

### 2.9 MiRNA-seq and bioinformatics analysis

Total RNA was extracted from samples (exo and exo-T) using SeraMir Exosome RNA Column Purification Kit (SystemBio, Palo Alto, CA, USA). After DNA digestion, RNA integrity and quality were confirmed by 1.5% agarose gel electrophoresis and a Nanodrop™ OneC spectrophotometer (Thermo Fisher Scientific Inc., MA, USA), respectively. Afterwards, the miRNA library was prepared using a KC-Digital™ small RNA Library Prep Kit for Illumina^®^ (Wuhan Seqhealth Co., Ltd., Wuhan, China). Next, the eluted cDNA library was size-selected with agarose gel electrophoresis and the ~160 bp bands were isolated, purified, and quantified by Qubit3.0, and finally sequenced on a HiSeq X-10 PE150 sequencer (Illumina, San Diego, CA, USA).

The miRNA differential expression was identified using the edgeR package (version: 3.12.1) between exo and exo-T groups. The miRNAs with a cutoff of a P-value < 0.05 and |Log2Fold-change| > 1 were considered differentially expressed. The target genes of differentially expressed miRNAs (DE-miRNAs) were predicted using RNAhybrid and miRanda v3.3a. Gene ontology (GO) and Kyoto Encyclopedia of Genes and Genomes (KEGG) enrichment analysis of targeted genes was conducted using KOBAS software (version: 2.1.1); the pathways meeting a corrected P-value cutoff of 0.05 were defined as significantly enriched pathways in the target genes of DE-miRNAs.

### 2.10 Western blot analysis

Proteins were extracted in RIPA lysis buffer supplemented with protease inhibitors. The concentration of lysed proteins was measured using a BCA kit (Thermo Fisher Scientific Inc., MA, USA). Then, proteins were separated via 10% or 12% SDS-PAGE and transferred onto PVDF membranes (Sigma, St. Louis, MO, USA). The membranes were blocked by Quick Blocking Solution (Biyuntian Biotechnology Co., Ltd., Shanghai, China) for about 10–15 min before incubation with primary antibodies and secondary antibodies. An ECL kit (Dalian Meilun Biotechnology, Dalian, China) was used to detect the blots, and the target protein signals were quantified using ImageJ software.

### 2.11 Statistical analysis

All statistical data were analyzed using GraphPad Prism 9.3.1. A t-test was performed to analyze statistical significance between two groups. One-way ANOVA was used to analyze multiple-group comparisons. Results were expressed as mean ± SD. A P-value < 0.05 was taken to indicate significant difference.

## 3 Results

### 3.1 Characterization of TNF-α-stimulated EC-derived exosomes and cellular uptake in Mϕ

**Figure 1:**
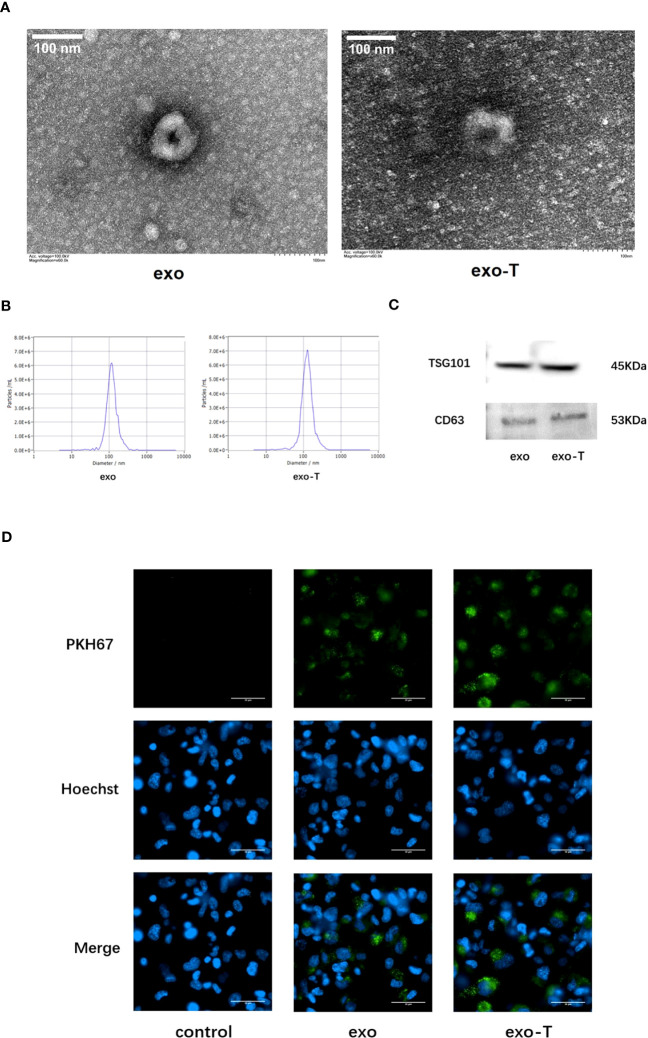
Characterization of exosomes from ECs with or without TNF-α stimulation and cellular uptake in Mϕ. **(A)** TEM images of exo and exo-T. **(B)** NTA results showing exosome size distribution and concentration. **(C)** Exosome markers CD63 and TSG101 were detected by immunoblot. **(D)** PKH67-labeled exo/exo-T (green) were incorporated into Mϕ. No green fluorescence was presented in the control with the same volume of PBS. Nuclei were stained with Hoechst (blue).

Human umbilical vein ECs were incubated for 24 h with or without TNF-α in the exosome-depleted serum medium for exosome culture. Transmission electron microscopy (TEM) images showed that both group of exosomes had a typical cup-like morphology with high edge density and low inner density (**Figure 1A**). The size distribution and concentration of exosomes were determined using NTA. The result showed that the concentration of exo/exo-T was 9.4E+9 and 1.4E+10 particles/mL, and the average size was 125.4 and 134.8 nm, respectively (**Figure 1B**). The presence of exosome markers CD63 and TSG101 was confirmed by immunoblot protein analysis in both groups (**Figure 1C**). Mϕ were incubated with PKH67-labeled PBS/exo/exo-T. The result indicates that PKH67-labeled exo/exo-T (green) were incorporated into Mϕ (**Figure 1D**), which proved that EC-derived exosomes were transferred into Mϕ and internalized rather than simply remaining on the cell surface.

### 3.2 Exo-T affected macrophage polarization and lipid deposition

**Figure 2:**
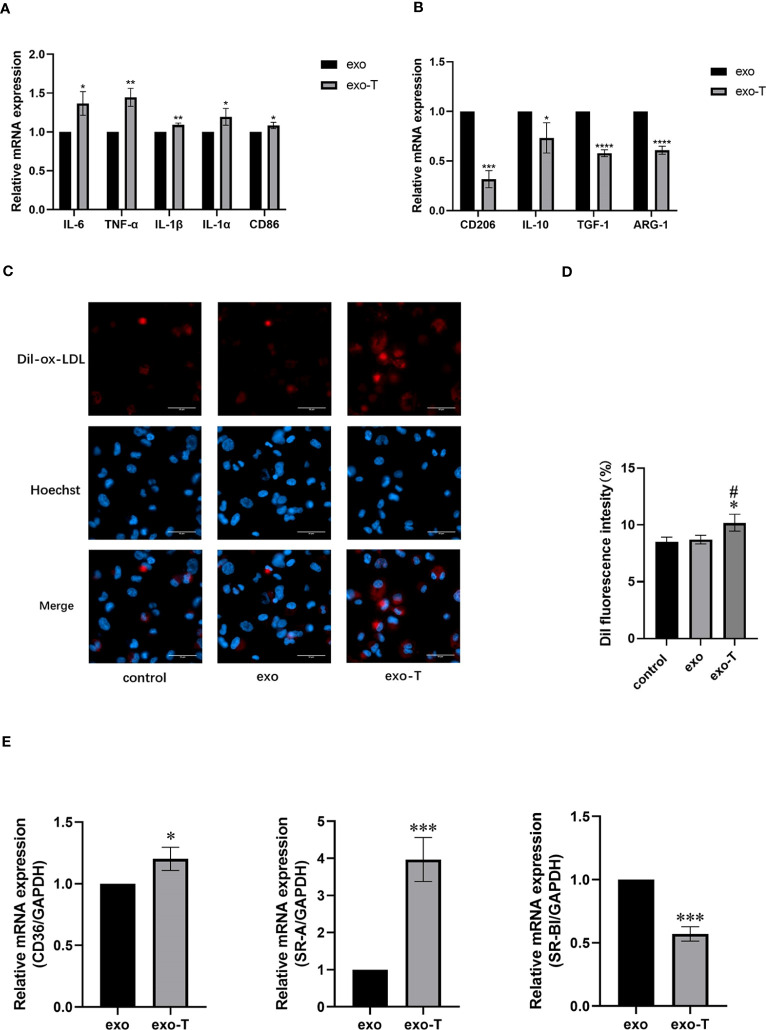
Exo-T skewed Mϕ to the M1 subtype and exacerbated Mϕ lipid deposition. Bar graph shows the relative mRNA expression levels of M1 signature genes IL-6, TNF-α, IL-1b, IL-1a, and CD86 **(A)** and M2 signature genes IL-10, TGF-1, ARG-1, and CD206 **(B)**;(n=3). **(C)** Mϕ lipid deposition was quantified by Dil-ox-LDL labeling (red). Nuclei were stained with Hoechst (blue);(n=3). **(D)** Bar graphs show quantitation results of fluorescence intensity;(n=3). **(E)** CD36, SR-A, and SR-BI relative mRNA expression was analyzed by RT-qPCR;(n=3). Data are presented as the mean ± SD from three independent experiments. *P < 0.05, **P < 0.01, ***P < 0.001, ****P < 0.0001 vs the exo group. #*P* < 0.05 vs the control group.

Activated macrophages differentiate into distinct phenotypes, which exhibit divergent functions. To investigate the effect of exo-T on Mϕ polarization, mRNA expression levels of macrophages co-cultured with exo-T were measured using qRT-PCR. Compared with the exo group, the mRNA expression of either M1 signature cytokines IL-6, TNF-α, IL-1b, and IL-1a or characterized gene CD86 in the exo-T group was significantly elevated (**Figure 2A**), while expression of M2 signature anti-inflammatory mediators IL-10, TGF-1, ARG-1, and CD206 was attenuated (**Figure 2B**), indicating that the influence of exo-T may play an important role in macrophage M1 polarization. One of the most striking hallmarks of macrophage dysfunction is lipid accumulation. To examine the effect of exo-T on Mϕ lipid deposition, ox-LDL uptake was evaluated by Dil-ox-LDL immunofluorescent staining (red). After the exposure to exo-T, the intracellular Dil-ox-LDL content increased dramatically more than that in the control or exo group (**Figures 2C, D**). As shown in **Figure 2E**, exo-T promoted the mRNA expression of CD36 and SR-A, while suppressing SR-BI. These results imply that exo-T enhances macrophage lipid accumulation.

### 3.3 Exo-T affected macrophage apoptosis but not cell migration

**Figure 3:**
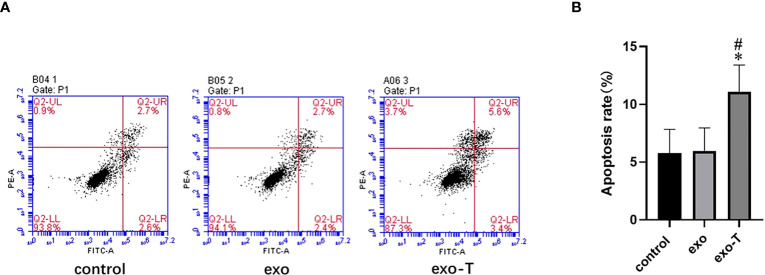
Exo-T promoted Mϕ apoptosis. **(A)** Mϕ apoptosis determined by flow cytometry (flow-cytometric plots);(n=4). **(B)** Apoptotic macrophage rate quantification;(n=4). Data are presented as the mean ± SD from four independent experiments.*P < 0.05 vs the exo group. #*P* < 0.05 vs the control group.

**Figure 4:**
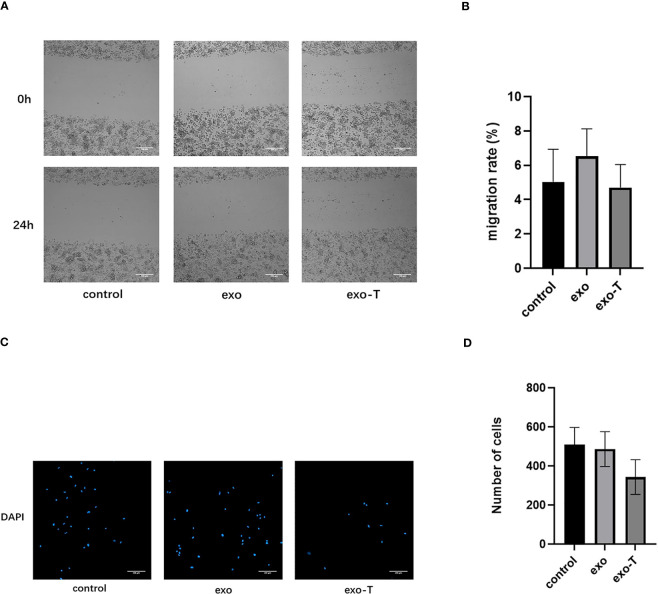
Exo-T might not significantly affect Mϕ migration. **(A)** Images of wound healing were captured at 0 and 24 h;(n=3). **(B)** Quantification of migration rate;(n=3). **(C)** The bottom of the transwell chambers was visualized using a High Content Imaging System (PerkinElmer). Nuclei were stained with DAPI (blue);(n=3). **(D)** Number of cells was counted by Harmony 4.5 software;(n=3).Data are presented as the mean ± SD from three independent experiments.

In addition to Mϕ polarization and lipid uptake, cell apoptosis and migration are also features for concern in atherogenic Mϕ. To investigate the role of exo-T in Mϕ apoptosis, the apoptosis rates were evaluated by flow cytometry with FITC annexin V/propidium iodide (PI) double staining. As shown in **Figure 3**, the Mϕ treated with exo-T underwent apoptosis more than Mϕ cultured with PBS or exo, which revealed that exo-T promotes Mϕ apoptosis. Next, we further explored the role of exo-T in Mϕ migration. Wound healing and transwell assays were carried out to assess the lateral and longitudinal migration capacity. As shown in **Figure 4**, there were no remarkable differences in wound closure ratios or numbers of migrating cells among the three groups. These results indicate that exo-T might have no significant effect on Mϕ migration.

### 3.4 MiRNA-seq and bioinformatics analysis indicates exo-T might activate Mϕ through MAPK/NF-κB pathways

**Figure 5:**
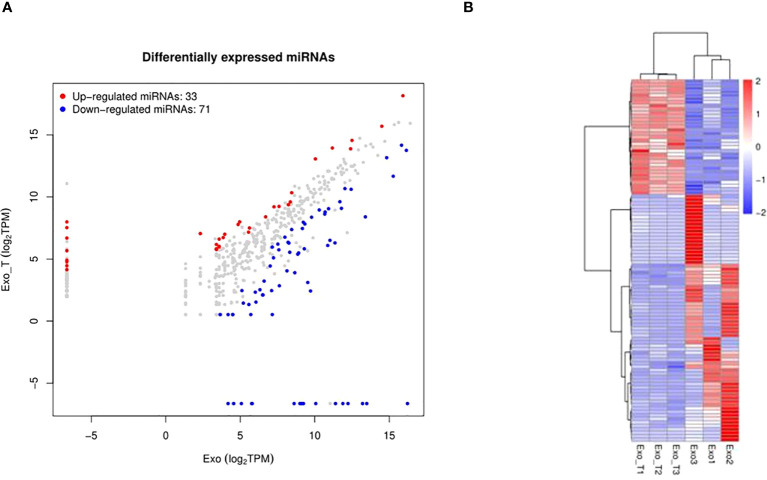
MiRNA-sequencing between exo and exo-T for comparison. **(A)** Scatter plot visually showing significantly differentially expressed miRNAs between the comparison groups. **(B)** Heat map analysis of differentially expressed miRNA; (n=3).

**Figure 6:**
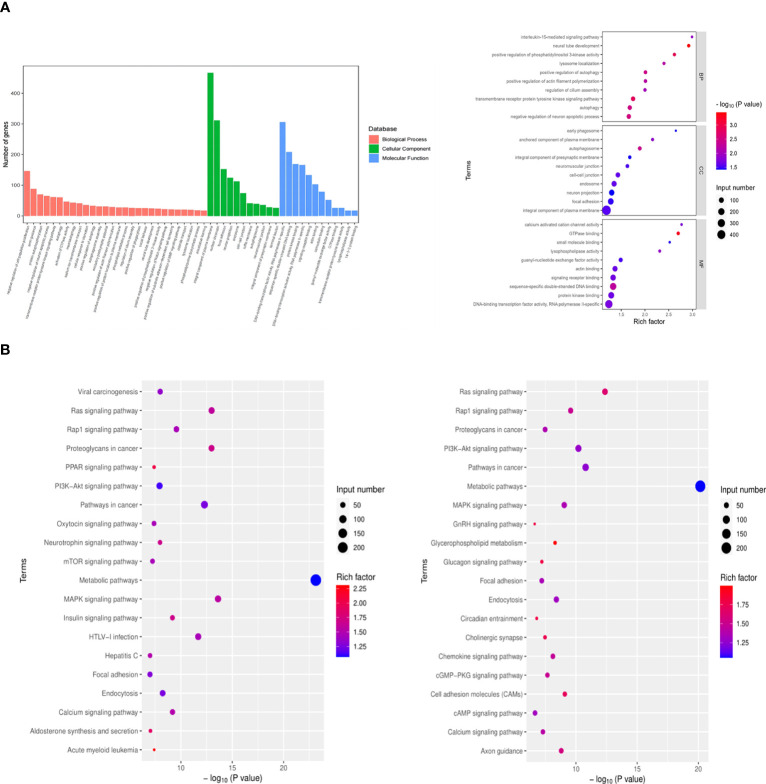
GO biological processes and KEGG pathways significantly enriched with genes targeted by DE-miRNAs. **(A)** GO enrichment analysis. **(B)** KEGG enrichment analysis. Input number represents the number of differential genes. Rich factor is the ratio of gene numbers to all gene numbers annotated in the KEGG pathway.

**Figure 7:**
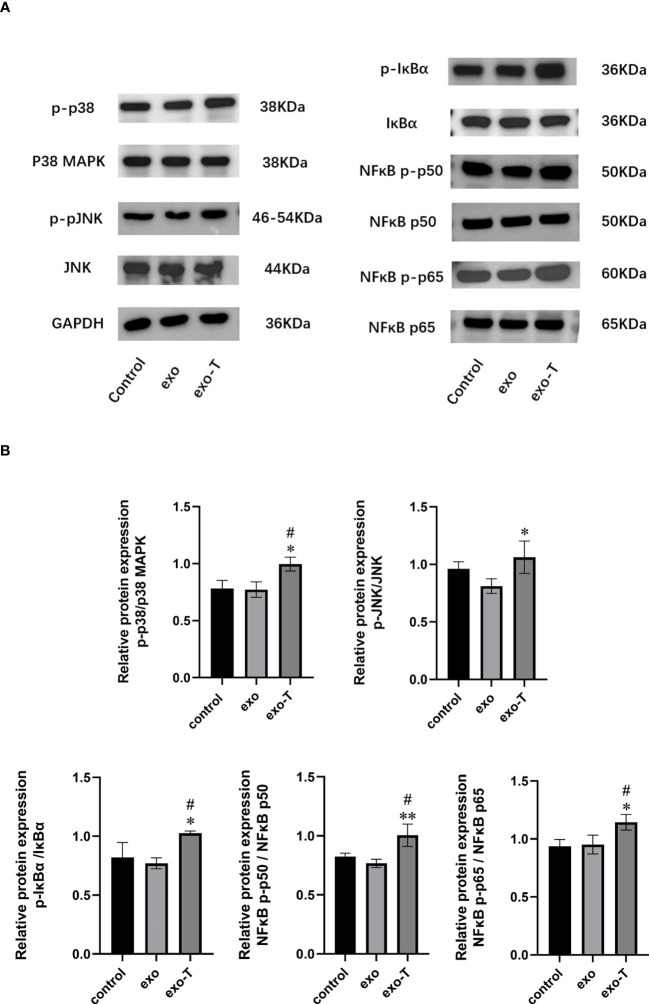
MAPK/NF-κB-related protein phosphorylation levels were improved in Mϕ following treatment with exo-T. **(A)** Band labels are as identified by western blot;(n=3). **(B)** The bar graph represents quantification of immunoblot bands using ImageJ software;(n=3). Data are presented as the mean ± SD from three independent experiments.*P < 0.05, **P < 0.01 vs the exo group. #*P* < 0.05 vs the control group.

Exosomes, acting as intercellular mediators, transfer messages to alter the gene expression and functions of distant cells, especially through functional miRNAs. Based on the result of exo and exo-T miRNA-Seq analysis, DE-miRNAs were defined when FDR < 0.05 and |log 2 FC| > 1. A total of 104 significantly DE-miRNAs were observed in the scatter plot, 33 upregulated miRNAs and 71 downregulated miRNAs (**Figure 5A** and **Supplementary Table 2**). The differential expression of miRNAs is further shown on a heatmap (**Figure 5B**). Then, we identified the DE-miRNA target genes from RNAhybrid and miRanda databases. GO enrichment analysis was performed on these target genes of 104 significantly DE-miRNAs, which identified the top 10 GO terms in each ontology as shown in **Figure 6A**. The mainly enriched BP (biological process) terms were “neural tube development”, “transmembrane receptor protein tyrosine kinase signaling pathway”, and “positive regulation of phosphatidylinositol 3-kinase activity”, and the MF terms (molecular function) included “GTPase binding”, “sequence-specific double-stranded DNA binding”, and “lysophospholipase activity”. In addition, CC (cellular component) terms included “autophagosome”, “anchored component of plasma membrane”, and “cell–cell junction”. Next, KEGG enrichment analysis was used to determine the pivotal signaling pathways involved in target genes of upregulated/downregulated DE-miRNAs. The bubble chart displays the top 20 KEGG pathways, significantly enriched in metabolic pathways, MAPK signaling pathway, cancer, and RAS signaling pathway (**Figure 6B**). MAPK/NF-κB pathways have been reported to be classical pathways for macrophage activation. To further confirm the essential role of MAPK/NF-κB pathways, the influence of Mϕ treated with exo-T was determined by western blot. As shown in **Figure 7**, compared with the other two groups, macrophage protein expression levels of p-p38/p38, p-JNK/JNK, p-IκBα/IκBα, p-p50/p50, and p-p65/p65 were markedly increased after exposure to exo-T. Together, these results further demonstrate that MAPK/NF-κB pathways might be involved in the activation of macrophages by exo-T.

## 4 Discussion

Intercellular communication is essential for almost all physiological and pathological processes (18). The development of atherosclerosis is a highly dynamic and intricate process with the participation of various cell types (19). ECs, the primary defense barriers in the vasculature, actively regulate the basic physiologic metabolism and maintain cardiovascular homeostasis. And macrophages, as innate immune-inflammatory cells, also play an important role in atherosclerotic plaque formation (16). Meanwhile, exosomes are regarded as the new participant of cell-to-cell communication in the atherosclerotic microenvironment (20). Thus, this study aimed to investigate whether exosomes from TNF-α-stimulated ECs have some effects on macrophages.

Exosomes are now known to be secreted by virtually all cells (21). ECs exhibit a potent paracrine ability that can release exosomes in different pathophysiological states (12). According to the requirements suggested by the International Society for Extracellular Vesicles (ISEV), we interconverted the morphology, particle diameter, concentrations, and protein markers of exosomes using TEM, NTA, and western blot (22). Then, we observed that PKH67-labeled exo/exo-T were taken up by macrophages. It was confirmed that exosomes from ECs acted as messengers of intercellular communication, which laid the foundation for further experiments (8).

In response to various stimuli, macrophages exhibit considerable phenotypic plasticity, interconverting between classically (M1) or alternatively (M2) activated macrophages. M1 macrophages feature a stable pro-inflammatory phenotype that secretes the factors IL-1b, IL-6, and TNF-α; M2 macrophages with anti-inflammatory properties express CD206 and ARG-1 characteristically and secrete the factors TGF-1 and IL-10. Jansen et al. (23) have shown that exosomes from high glucose-pretreated ECs facilitate M1 macrophage infiltration and the expression of adhesion proteins. He et al. (24) observed that exosomes from ox-LDL-treated ECs promote the polarization of M1 macrophages and release of inflammatory cytokines. In the two above-mentioned studies, it was suggested that the macrophage phenotype could be regulated by EC-exo from different microenvironments. In this study, we observed that exo-T also polarized macrophages to the M1 subtype and contributed to cell inflammation.

A change in macrophage lipid metabolism is also a key feature of atherosclerotic initiation and progression. The extent of Mϕ lipid deposition is closely associated with the balance of lipid uptake and retrograde transport, which is influenced by lipoprotein receptors, such as CD36, scavenger receptors (SR-A, SR-BI), etc. In this study, we found that exo-T enhanced Mϕ lipid deposition, which may be correlated with CD36 and SR-A activation and SR-BI inhibition.

Besides Mϕ phenotype and lipid metabolism, the numbers of macrophages in the atherosclerotic lesion also deserve attention, which are affected by many factors (25). For example, macrophage proliferation *in situ* and monocyte recruitment increase macrophage number, while macrophage apoptosis and migration counteract the former.

We demonstrated that exo-T promotes Mϕ apoptosis. And our study revealed that exo-T might show some trends to inhibit Mϕ migration, but have no significant differences. Meanwhile, in addition to promoting foam cell formation, prior studies have illustrated that CD36/SR-A activation also regulates Mϕ apoptosis by inflammatory response and attenuates Mϕ migration by cytoskeleton changes (26, 27). In this study, the former part’s results also displayed that exo-T enhances the expression of CD36 and SR-A. Our observations were basically in accordance with results from previous studies.

Although the impact of Mϕ function was investigated in this study, the possible mechanistic pathways of exo-T involvement in these effects require further study. Increasing evidence indicates that microRNAs carried by exosomes play an important role in regulating gene transcription of the recipient cells in cell-to-cell communication, considered to be a new type of molecule that regulates biological processes, including inflammation, angiogenesis and apoptosis (28–30). In this study, analysis of exo/exo-T microRNA-Seq revealed 104 significantly DE-miRNAs. And then, through KEGG enrichment analysis searching the signaling pathways involved in the target genes of DE-miRNAs, we found that the pathways were predominantly enriched in metabolic pathways, MAPK signaling pathway, etc. Other scholars have shown that exosomes from TNF-α-stimulated ECs triggering the inflammatory response might involve the NF-κB pathway (31, 32). And studies have confirmed that MAPK/NF-κB signaling pathways contribute to atherosclerotic lesion formation, including regulating Mϕ phenotype, lipid deposition, apoptosis, migration, etc. (33, 34). Our immunoblot results indeed are in line with prior studies and bioinformatic analysis outcomes. Thus, this study supports that exo-T might activate macrophages through MAPK/NF-κB signaling pathways.

As demonstrated recently for the MAPK/NF-κB signaling pathways, their broad inhibition is extremely risky, because they play generalized roles in inflammation and immunity and serve as a common pathway for numerous pathophysiological mechanisms (35). Accumulating evidence shows that localized anti-inflammatory therapy reducing host defense is significantly better than systemic therapy (36). Due to elevated levels of EC-exo correlating with pro-inflammatory and pathologic conditions, Zhang et al. (13) provided evidence that PAK4 suppresses EC-exo generation, which might represent a novel approach to ameliorate inflammatory diseases. Therefore, further study may focus on designing an exosome inhibitor targeting blockage of key site activation through researching multifaceted potential pathomechanisms and sites of action.

Accumulating evidence indicates that exosomes have a double-edged sword regulatory role to decrease/facilitate atheroma formation under different experimental conditions or even at different pathological stages of atherosclerosis. And exosomes regulate target organs through multiple pathways and sites of action, whereas our study only included a possible single pathway. Thus, combining other regulatory mechanisms, further investigation will be based on comprehensive consideration of the overall disease mechanism. As indicated by Mahmoud et al. (37), a TNF-α-stimulated EC-exo is in fact a paradoxical vehicle carrying cellular messages. Although conventional wisdom suggests that exo-T seems to be more like a pathogenic mediator and increases in inflammatory vascular disease, indeed, it may play a dual role, which has been well established in sepsis. The number and function of exosomes may vary depending on the microenvironment, pathogenetic mechanisms, and sites of atheroma formation. Thus, the exosome containing unique prognostic information is still needed for further study, which may improve risk-stratification of atherosclerosis patients and provide improved treatment strategies.

In conclusion, this study demonstrated that exosomes from TNF-α-stimulated ECs are taken up by Mϕ, skew Mϕ toward an M1 phenotype, and enhance Mϕ lipid deposition and apoptosis, but do not significantly affect Mϕ migration. And exosomes from TNF-α-stimulated ECs promote the phosphorylation of MAPK and NF-κB-related proteins in the macrophages, which indicates that macrophage activation might be associated with the MAPK and NF-κB pathways.

## Data availability statement

The datasets presented in this study can be found in online repositories. The names of the repository/repositories and accession number(s) can be found below: GSE225467 (GEO).

## Author contributions

PZ and FH designed this study. WL, FH, and YY performed the experiments and analyzed data. QL, ZL, WZ, and BL provided experimental support. WL wrote the manuscript. PZ, FH, and YY reviewed and revised the manuscript. All authors contributed to the article and approved the submitted version.
