# Supplementary material for: Endothelial exosomes work as a functional mediator to activate macrophages
Source: Front Immunol. 2023 Jul 28;14:1169471. doi: 10.3389/fimmu.2023.1169471 (PMC10416261; doi:10.3389/fimmu.2023.1169471)
Supplement: Supplementary file 1 [file DataSheet_1.pdf]

Supplementary Material

Endothelial exosomes work as a functional mediator to activate macrophages

Wenwen Lin[12345 †], Feng Huang[12345 †], Yin Yuan[12345 †], Qiaowei Li[12345], Zhong Lin[21345], Wenqing Zhu[21345], Binbin Lin[21345], and Pengli Zhu[12345*]

[1]Department of Geriatric Medicine, Fujian Provincial Hospital, Fuzhou, China;

[2]Shengli Clinical Medical College, Fujian Medical University, Fuzhou, China;

[3]Fujian Provincial Institute of Clinical Geriatrics, Fuzhou, China;

[4] Fujian Provincial Center of Geriatrics, Fuzhou, China;

[5]Fujian Provincial Key Laboratory of Geriatrics, Fuzhou, China.

†The authors contributed equally to this work and share first authorship.

*** Correspondence:**
Pengli Zhu*
zpl7755@hotmail.com.

Supplementary Table 1. RT-PCR primers

Primer names	5’- 3’ Sequences
IL-6-F(homo)	AAGCCAGAGCTGTGCAGATG
IL-6-R(homo)	CTGGCATTTGTGGTTGGGTC
TNF-a-F(homo)	TCCTCTCTGCCATCAAGAGC
TNF-a-R(homo)	AGTAGACCTGCCCAGACTCG
IL-1β-F(homo)	AGCTACGAATCTCCGACCAC
IL-1β-R(homo)	CGTTATCCCATGTGTCGAAGAA
IL-1α-F(homo)	CTGGGAAACTCACGGCACTA
IL-1α-R(homo)	ACGCCTGGTTTTCCAGTATCT
CD86-F(homo)	TACACGGTTACCCAGAACCT
CD86-R(homo)	CCGCGTCTTGTCAGTTTCCA
CD206-F(homo)	TGGTGAACGGAATGATTGTGTAG
CD206-R(homo)	GGTCCATCTTCCTTGTGTCAG
IL-10-F(homo)	AGCTGAGAACCAAGACCCAGA
IL-10-R(homo)	ACTCATGGCTTTGTAGATGCCTT
TGF-1-F(homo)	GAGCCCTGGACACCAACTAT
TGF-1-R(homo)	AAGTTGGCATGGTAGCCCTT
Arg1-F(homo)	GGAAGACACCAGAAGAAGTAACTC
Arg1-R(homo)	GGTTAAGGTAGTCAATAGGCTTGT
CD36-F(homo)	GCTTAATGAGACTGGGACCAT
CD36-R(homo)	CACCACACCAACACTGAGTAA
SR-A-F(homo)	GCTCAACTCCTGAAGTGGGA
SR-A-R(homo)	TCTGTGTCCATGAGGTTGGC
SR-BI-F(homo)	CTCACTTCCTCAACGCTGAC
SR-BI-R(homo)	GGACCACAGGCTCAATCTTC
GAPDH-F(homo)	GGTGTGAACCATGAGAAGTATGA
GAPDH-R(homo)	GAGTCCTTCCACGATACCAAAG

Supplementary Table 2. The list of 104 significantly DE-miRNAs

miRNA	logFC
novel_118	7.764736221
novel_124	7.31469714
novel_126	6.481327907
hsa-miR-582-3p	5.460892659
hsa-miR-330-3p	4.723223114
hsa-miR-23a-5p	4.7047556
hsa-miR-4725-3p	4.317629507
hsa-miR-4645-3p	4.040434606
hsa-miR-1180-3p	3.531282388
hsa-miR-125a-3p	3.4071556
hsa-miR-3615	3.124193285
hsa-miR-155-5p	3.015083523
hsa-miR-3200-3p	2.982397337
hsa-miR-146a-3p	2.937581892
hsa-miR-99b-3p	2.903834623
hsa-miR-935	2.873458648
hsa-miR-1271-5p	2.812676289
hsa-miR-146a-5p	2.768489158
hsa-miR-196a-5p	2.75818768
hsa-miR-4449	2.74319252
hsa-miR-18a-5p	2.692897544
hsa-miR-126-3p	2.235023083
hsa-miR-421	1.960067864
hsa-miR-24-3p	1.951676221
hsa-miR-196b-5p	1.945473997
hsa-miR-503-5p	1.875102469
hsa-miR-361-3p	1.575649301
hsa-miR-324-5p	1.563701874

miRNA	logFC
hsa-miR-139-5p	1.450424286
hsa-miR-23a-3p	1.412747313
hsa-miR-424-5p	1.141595446
hsa-miR-574-3p	1.104389155
hsa-let-7i-5p	1.085532228
hsa-miR-376a-3p	-1.167135208
hsa-miR-144-3p	-1.323656609
hsa-miR-146b-5p	-1.334612259
hsa-miR-376b-3p	-1.335990176
hsa-miR-494-3p	-1.38267119
hsa-miR-142-3p	-1.391590283
hsa-miR-148a-3p	-1.440752786
hsa-miR-142-5p	-1.588523783
hsa-miR-411-5p	-1.600259084
hsa-miR-223-3p	-1.717079825
hsa-miR-199a-3p	-1.837080794
hsa-miR-199b-3p	-1.837538871
hsa-miR-451a	-1.907866628
hsa-miR-335-5p	-1.942460557
hsa-miR-199a-5p	-1.995010695
hsa-miR-127-5p	-2.013868149
novel_8	-2.053487374
hsa-miR-100-5p	-2.071167281
hsa-miR-486-5p	-2.078690818
hsa-miR-194-5p	-2.105928849
hsa-miR-493-3p	-2.284922466
novel_9	-2.665273399
hsa-miR-125b-5p	-2.681686197

miRNA	logFC
novel_25	-2.725933379
hsa-miR-190a-5p	-2.736891183
hsa-miR-296-5p	-3.051313567
hsa-miR-708-3p	-3.242568609
hsa-miR-203a-3p	-3.356819997
hsa-miR-205-5p	-3.392531088
hsa-miR-337-3p	-3.432026001
hsa-miR-215-5p	-3.562258991
hsa-miR-145-5p	-3.606831834
hsa-miR-138-5p	-3.804660784
hsa-miR-182-5p	-3.892022185
hsa-miR-200c-3p	-3.974149919
hsa-miR-873-5p	-3.974149919
hsa-miR-4521	-3.99470352
hsa-miR-34c-5p	-4.009627085
hsa-miR-135a-5p	-4.052215222
hsa-miR-9-5p	-4.054306375
hsa-miR-145-3p	-4.085578473
hsa-miR-490-3p	-4.219144319
hsa-miR-34b-5p	-4.479811601
hsa-miR-200b-3p	-4.562600995
hsa-miR-219a-5p	-4.59316921
hsa-miR-199b-5p	-4.682696913
hsa-miR-141-3p	-4.793788426
hsa-miR-204-5p	-4.923412494
hsa-miR-100-3p	-5.019180854
hsa-miR-200a-3p	-5.228376509
hsa-miR-222-5p	-5.241979457

miRNA	logFC
hsa-miR-143-3p	-5.275527815
hsa-miR-338-3p	-5.293050158
hsa-miR-9-3p	-5.53764436
hsa-miR-183-5p	-6.168117799
hsa-miR-96-5p	-6.272759157
hsa-miR-708-5p	-6.322300404
hsa-miR-124-3p	-6.595347092
hsa-miR-143-5p	-7.150394556
novel_27	-8.702108763
novel_41	-8.879307679
hsa-miR-219a-2-3p	-8.971598176
novel_22	-8.985358589
novel_30	-9.190512643
novel_11	-9.788314387
novel_31	-11.10078441
novel_7	-11.63509645
novel_60	-11.97068066
novel_65	-13.20371449
novel_64	-13.27446263
novel_1	-16.22481727
